# Quality Markers for Astragali Radix and Its Products Based on Process Analysis

**DOI:** 10.3389/fphar.2020.554777

**Published:** 2020-12-18

**Authors:** Yuntao Dai, Dongbo Wang, Manjia Zhao, Lihua Yan, Chao Zhu, Pengyue Li, Xuemei Qin, Rob Verpoorte, Shilin Chen

**Affiliations:** ^1^ Institute of Chinese Materia Medica, China Academy of Chinese Medical Sciences, Beijing, China; ^2^ College of Medicine and Nursing, Dezhou University, Shandong, China; ^3^ China Modern Research Center for Traditional Chinese Medicine, Shanxi University, Shanxi, China; ^4^ Natural Products Laboratory, Institute of Biology, Leiden University, Leiden, Netherlands

**Keywords:** astragali radix, quality control, process analysis, quality markers, fingerprint analysis

## Abstract

Due to the complex nature of traditional medicines, quality control methods need to cover two aspects: compliance of raw materials with quality standards and process control. Astragali radix (AR), the roots of *Astragalus mongholicus* Bunge, was selected in this study as an example of a widely used traditional medicine in various formulations. Astragaloside IV (AG IV) and calycosin 7-*O*-β-D-glucoside (CG) are used as the markers for the quality control of AR and its products in the Chinese Pharmacopoeia. However, in the raw materials, malic acid esters of the CG and acetate esters of the astragaloside are easily decomposed into CG and AG IV during storage and processing of AR to make extracts for various preparations. The thermal stability of the isoflavonoids and astragalosides in decoction was studied. The level of CG and astragalosides (AG I/AG II/AG IV) was strongly affected by prolonged heat during processing, while calycosin was stable in the conditions. Also the major astragalosides in AR could fully converted into AG IV which eventually reaches a stable level under certain conditions. With calycosin and AG IV as marker components, practical, reproducible, and precise methods were established and applied to the quality analysis of AR from its raw materials to its intermediates and products. This study demonstrates that a full chemical profiles analysis of the whole manufacturing process (from “raw materials—intermediates/extracts—final product”) is important to identify quality markers (Q-markers) and even to establish proper analysis methods for traditional Chinese medicine products.

## Introduction

Astragali radix (AR) (Huang Qi in Chinese) is derived from the dried roots of *Astragalus mongholicus* or *A. membranaceus* (Fisch.) Bge (National Commission of Chinese Pharmacopoeia, 2015). For more than 2,000 years it is one of the most widely used Chinese herbal medicines and also functional food for reinforcing “Qi” (vital energy). It’s traditionally used for revitalizing, tonifying, skin reinforcement diuretic, abscess-draining, and tissue-generative purpose (National Commission of Chinese Pharmacopoeia, 2015). Modern pharmacological studies have demonstrated that AR possesses a variety of biological activities, such as immunomodulating (Jin et al., 1994), anti-oxidative (Lee et al., 2005), and cardiovascular protective function in diabetic nephropathy (Fu et al., 2014). AR is widely used in different complex formulations, and there are almost 150 traditional Chinese patent medicines (TCPMs) containing AR described in the Chinese Pharmacopoeia (2015 version) (National Commission of Chinese Pharmacopoeia, 2015).

The TCPMs that contain AR include granules, capsules, tablets, syrups, plasters/concentrated decoctions, and so on. The manufacturing processes of these products must at least include the extraction of AR pieces with hot water, followed by concentration of the decoction (National Commission of Chinese Pharmacopoeia, 2015). During the processing, from raw materials to concentrated extract, conversions of constituents may occur due to e.g., high temperature, and prolonged contact with air, pH and water (Lin et al., 2000; Chu et al., 2014). For a proper quality control ensuring reproducible end products, Q-markers are required that have a stable level during the whole production process. The Q-markers should be chemically stable and its levels can be followed during the whole manufacturing process. So whole process (i.e., from “raw materials—intermediates/extracts—final product”) analysis is very necessary to identify Q-markers.

Isoflavonoids and triterpene saponins are important active compounds in AR. Astragaloside IV (AG IV) and calycosin 7-*O*-β-D-glucoside (CG) are used as the marker substances for the quality control of AR and its products in the Chinese Pharmacopoeia (National Commission of Chinese Pharmacopoeia, 2015). However, it has been reported that calycosin 7-*O*-β-D-(6″-malonyl) glucoside (CGM) is unstable during sample preparation and may degrade into CG (Lin et al., 2000). Astragaloside I (AG I), isoastragaloside I (iAG I), astragaloside II (AG II), and acetylastragaloside I (acetyl AG I) were also shown to be unstable and to be converted into AG IV under basic conditions (Chu et al., 2014). These observations imply that the CG and AG IV contents detected in AR are the results of chemical transformations during manufacturing process and sample preparation, and thus their levels are affected by the degree of transformation. Therefore, the present quality analysis methods based on AG IV and CG as Q-markers of AR and its products should be reconsidered. Previous studies on AR, have identified the major small molecules and different quantification methods have been developed for AR and its products (Qi et al., 2006; Song et al., 2007; Qi et al., 2008; Song et al., 2008; Zhao et al., 2018). However, the stability of the levels of Q-makers during the whole manufacturing process and sample preparations were neglected. Specific studies on the stability of the levels of these constituents and their possible chemical conversions during manufacturing processes and sample preparation are essential to guarantee the accuracy of the quality control methods.

**FIGURE 1:**
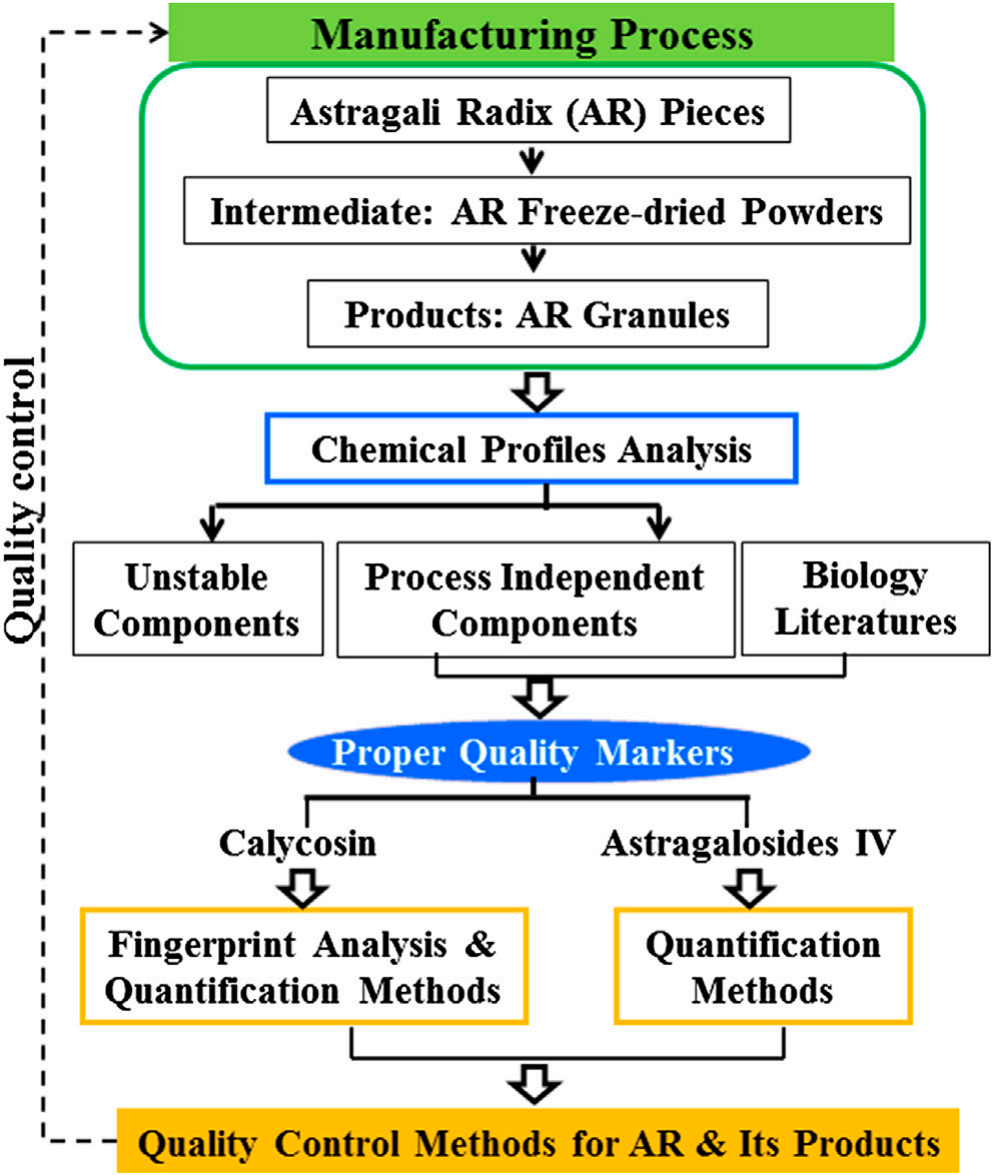
Flow chart of integrated strategy for screening process independent markers and establishing quality control methods for raw materials and its products with Astragali Radix.

This study aims to analyze the stability of the major components of AR during the manufacturing process to identify proper Q-markers for AR, and then develop a practical analytical method for the quality control of AR and its preparations based on these markers. The analysis of the stability of the levels of major components of AR was done by following the changes of chemical profiles throughout the manufacturing process (i.e., from “pieces—freeze-dried powders of extracts—granules”) (Figure 1). To achieve a proper quality control protocol, fingerprint analysis of isoflavonoids including quantitation of calycosin content was established, as well as an optimized method for the determination of AG IV content in AR and its products. This study demonstrates how to develop quality control methods for TCPMs that are independent of processing in the whole production chain from raw materials to its final products. The Q-markers can be accurately detected in raw materials to terminal products, which provides methods for the whole chain analysis of traditional Chinese medicine (TCM) and facilitates the traceability analysis of the quality of TCM.

## Materials and Methods

### Chemicals, Materials and Reagents

Pieces, freeze-dried powders of extracts, and formula particles (the final formulated product in the form of granules, batch NO. 406209L, 1406002, 1506027, 1601057, 6101141, 5090891) of AR were obtained from Tianjiang Yifang Company, China. A voucher specimen (RA-YP-1∼RA-YP-12; RA-DGF-1∼RA-DGF-12; RA-PFKL-1∼RA-PFKL-6) were deposited in the herbarium of the Institute of Chinese Materia Medica, China Academy of Chinese Medical Sciences. The dosage forms and batch numbers used are listed in Table 1. Reference compounds, including astragaloside IV (AG IV, S1, CHB170727), calycosin 7-*O*-β-D-glucoside (CG, F1, CHB161105), and calycosin (C, F7, CHB161104) were obtained from the National Institute for the Control of Pharmaceutical and Biological Products. Their purity, as determined by HPLC, was above 98%. The structures of these compounds are shown in Supplementary Figure S1. The quality of the AR pieces used met the requirement of the Chinese Pharmacopoeia (2015 version) (National Commission of Chinese Pharmacopoeia, 2015).

**TABLE 1:** The batch numbers, correlation coefficients to the mean chromatogram of pieces of Astragali Radix, and mean content of quality marker (calycosin and astragaloside IV) for pieces (YP-1–YP-12), freeze-dried powders (DGF-1–DGF-12), and formula granules (PFKL-1–PFKL-6) of Astragali Radix in this study. The mean content was calculated with the weight of target compounds divided by the weight of the samples.

No.	Batch number	Correlation coefficient	Content of quality marker (%) (*n* = 3)			
			Calycosin	RSD%	Astragaloside IV	RSD%
YP-1	AR-201504	0.980	0.007	3.85	0.06	0.93
YP-2	AR-201505	0.967	0.009	3.84	0.06	0.14
YP-3	AR-201506	0.989	0.015	2.83	0.06	0.71
YP-4	AR-201507	0.894	0.018	0.16	0.05	1.65
YP-5	AR-201508	0.906	0.023	0.24	0.05	0.72
YP-6	AR-201509	0.986	0.035	1.14	0.04	0.42
YP-7	AR-201510	0.969	0.016	0.99	0.05	1.27
YP-8	AR-201511	0.979	0.007	0.17	0.05	0.67
YP-9	AR-201512	0.976	0.007	4.26	0.05	1.92
YP-10	AR-201504	0.983	0.007	2.93	0.04	1.70
YP-11	AR-201505	0.943	0.005	0.00	0.06	1.65
YP-12	AR-201506	0.983	0.009	1.77	0.07	1.27
DGF-1	DG16009015	0.804	0.117	3.90	0.16	0.42
DGF-2	DG1609002	0.861	0.108	0.64	0.14	0.57
DGF-3	DG1609008	0.868	0.102	0.31	0.14	0.18
DGF-4	DG1611006	0.851	0.075	0.04	0.14	0.55
DGF-5	DG1609014	0.848	0.065	0.69	0.19	0.24
DGF-6	DG169011	0.786	0.066	0.27	0.14	0.34
DGF-7	DG1609007	0.768	0.096	1.34	0.12	0.69
DGF-8	DG1611003	0.807	0.065	2.21	0.13	0.34
DGF-9	DG1607007	0.867	0.078	0.95	0.13	0.29
DGF-10	DG1611007	0.795	0.072	3.08	0.15	0.63
DGF-11	DG1611002	0.794	0.174	0.08	0.12	1.36
DGF-12	DG1611001	0.795	0.058	2.21	0.10	2.74
PFKL-1	406209L	0.534	0.013	2.67	0.11	2.66
PFKL-2	1406002	0.591	0.030	1.51	0.13	0.96
PFKL-3	1506027	0.560	0.022	2.02	0.13	1.60
PFKL-4	1601057	0.584	0.028	0.93	0.13	1.68
PFKL-5	6101141	0.557	0.013	0.31	0.12	0.42
PFKL-6	5090891	0.544	0.016	0.71	0.12	2.98

HPLC-grade acetonitrile (Fisher Scientific, United States), Optima liquid chromatography–mass spectrometry (LC-MS)-grade formic acid (Fisher Scientific, Czech Republic), and pure water (Wahaha, China) were used as mobile phases. UniElut C_18_EC column (500 mg/6 ml) were bought from Acchrom Instrument (Beijing) Technologies Co., Ltd. Other reagents and chemicals were of analytical grade. All solvents and samples were filtered through 0.45-μm membrane filters (Jinteng, Tianjin, China) before being injected into the HPLC system.

### Preparation of Solutions

#### Preparation of Standards Solution

Reference compounds, including AG IV, CG, and C, were accurately weighed and dissolved in methanol to form 1 mg/ml stock solutions, and stored at 4°C for further use.

#### Sample Preparation for Isoflavonoid Analysis

Dried pieces of AR were milled to a homogeneous powder and then sieved through a No. 65 mesh listed in Chinese pharmacopeia. Samples of the powder were accurately weighed to a mass of 1 g, placed in a 50 ml centrifuge tube, and ultrasonicated (40 kHz, 500 W) with 15 ml of methanol for 30 min. After being centrifuged (at 3,000 × g) for 5 min, the methanol solution was collected and filtered. The residue was washed twice with 7.5 ml of methanol, ultrasonicated (40 kHz, 500 W) for 5 min, centrifuged (at 3,000 × g) for 5 min, and filtered again. The filtrates were combined, passed through a membrane filter (0.45 µm), and then a sample was injected into the HPLC system.

Freeze-dried powder and granule sample were accurately weighed to a mass of 0.3 and 0.8 g, respectively, and then placed in a 50 ml centrifuge tube and ultrasonicated (40 kHz, 500 W) with 20 ml of an aqueous solution containing 5% methanol for 10 min. After being allowed to cool, the methanol solution was filtered through a membrane filter (0.45 µm) and injected into the HPLC system for analysis.

#### Sample Preparation for Astragaloside IV Content Determination

Extraction and preparation of AR pieces was carried out as described in our previous study (Zhao et al., 2018).

Each sample of freeze-dried powder and formula granules was accurately weighed, 0.3 and 0.5 g, respectively, placed in a 50 ml centrifuge tube. After addition of 20 ml of aqueous solution containing 60% methanol, the sample was ultrasonicated (40 kHz, 500 W) 30 min. After adding 10 ml of ammonia, the aqueous methanol solution was centrifuged (at 3,000 × g) for 30 min and filtered. The residue was washed with a mixture of 2 ml of a 60% methanol solution and 1 ml of ammonia, and then centrifuged (at 3,000 × g) for 30 min. The filtrates were combined and loaded onto a solid-phase extraction column (SPE column, UniElut C18EC column, 500 mg/6 ml), which had been activated with 5.0 ml methanol. The SPE column was washed with 5.0 ml of water, and then eluted with 5.0 ml methanol. The methanol eluent was collected and filtered through a membrane filter (0.45 µm), and finally injected into the HPLC system for analysis.

### HPLC and MS Analysis Conditions

#### HPLC-DAD Conditions for Isoflavonoid Analysis

HPLC-DAD analyses were performed using a 1200 Series HPLC (Agilent)-DAD system. A YMC-Triart C18 column (250 × 4.6 mm. 5 µm) was used for the chromatographic separations. The mobile phase consisted of 0.1% formic acid in acetonitrile (A) and water with 0.1% formic acid (B), using a gradient elution of 5–20% A at 0–8 min, 20–25% A at 8–15 min, 25% A at 15–20 min, 25–40% A at 20–30 min, and 40–60% A at 30–40 min. The wavelength was set at 260 nm. The injection volume was 20 μl, and the flow rate was 1 ml/min.

#### HPLC-ELSD Conditions for Astragaloside Content Determination

HPLC with evaporative light scattering detector (HPLC-ELSD) analyses were performed using a 1200 Series HPLC (Agilent)-ELSD system (Alltech 2000 ES). A YMC-Triart C18 column (250 × 4.6 mm. 5 µm) was used for chromatographic separations. The mobile phase consisted of 0.1% formic acetonitrile (A) and water with 0.1% formic acid (B), using a gradient elution of 5–10% A at 0–5 min, 10–32% A at 5–10 min, 32–45% A at 10–30 min, 45–95% A at 30–35 min, and 95–5% A at 35–40 min. The injection volume was 20 μl, and the flow rate was 1 ml/min. ELSD was performed with nitrogen as the carrier gas at a flow rate of 2.5 L/min, and the nebuliser temperature was set to 100°C.

#### HPLC-Linear Trap Quadrupole-MS Analysis

To identify the major peaks in the HPLC-UV/ELSD chromatograms, the high-performance liquid chromatography system coupled with a high-resolution linear trap quadrupole Orbitrap mass spectrometry (Thermo Scientific, United States) was used. A full-scan model was employed, and the m/z range was set to 150–1,000. Electrospray ionization was done with the following conditions: a source heater temperature of 400°C, capillary temperature of 350°C, ion spray voltage of 3.5 kV, capillary voltage of 35 eV, gas flow rate of 35 arb, and auxiliary gas flow rate of 10 L/h. A data-dependent scan was used for tandem mass spectrometry (MS/MS).

### Method Validation

#### Calibration Curves and Linearity Range

A series of analyses were conducted to validate the performance of the methods, including determinations of their linearity, precision, repeatability, stability, and accuracy. The relative standard deviation (RSD) was used to evaluate the precision of the developed method. Methanol stock solutions of AG IV and C were prepared and diluted to appropriate concentration ranges for the construction of calibration curves. The calibration curve of C was constructed using its peak areas and its calculated concentrations. The calibration curve of AG IV was constructed by plotting the logarithm of the peak areas vs. the logarithm of its concentrations.

#### Precision, Repeatability, Stability, and Accuracy

Analyses of intra-day variations among six successive injections were chosen to determine the precision of the developed method. To confirm its repeatability, six different working solutions from the same sample were prepared and analyzed. The sample stability was determined by examining one sample at different times during one day, at 0, 1, 2, 4, 12, and 24 h. Over this period, the solution was stored at room temperature.

The recovery of the marker components was determined by spiking the same amount of reference standards into samples with known contents, and these were then extracted and analyzed as described in *Preparation of Solutions* and *HPLC and MS Analysis Conditions* sections. The recoveries were calculated with the following formulas: recovery (%) = (detected amount − original amount)/added amount × 100%; and RSD (%) = (SD/Mean) × 100.

### Stability Test of AR Decoction During Processing

#### Preparation of AR Decoction

Pieces of AR (100 g) were placed in 800 ml of water for 30 min at room temperature, and then refluxed for 1 h (at 100°C). Each decoction was filtered, and the residue was refluxed for another 30 min in 600 ml of water (at 100°C). The filtrates were combined and evaporated to a final volume of <500 ml under reduced pressure at a temperature not exceeding 50°C, and then the volume of the concentrated solution was adjusted to 500.0 ml in a volumetric flask using water. The solution was centrifuged at 12,000 rpm for 20 min prior to analysis of the supernatant.

#### Stability Test of AR Decoction in the Heating Process

The final AR decoction was separated into nine Eppendorf (EP) tubes, which were then closed and sealed with sealing film. Three of them were heated for 0, 0.5, and 2 h at 100°C, respectively, and the processed solutions were then cooled to room temperature, centrifuged at 12,000 rpm for 20 min, and analyzed by HPLC with DAD/ELSD.

#### Stability Tests of Aqueous Solution of Standards

Stability tests were conducted using pure compounds (AG II and AG IV) as representative of astragalosides and an aqueous solution of a mixture of main astragalosides (AG II, iAG II, AG I, and iAG I) in AR. Known amounts (10–50 μl) of the solutions of the target compounds were added to water prior to vortex mixing and centrifugation. The supernatant was divided into six tubes, which were closed and heated at 100°C for 2 h. The samples were then cooled to room temperature, filtered, and analyzed using HPLC-ELSD. These analyses were conducted as described in *HPLC and MS Analysis Conditions* section, and the peaks were identified using pure standard compounds and a high-resolution linear trap quadrupole Orbitrap, as described in *HPLC-Linear Trap Quadrupole-MS Analysis* section.

### Data Analysis

The software “Similarity Evaluation System for Chromatographic Fingerprint of TCM” published by GPC (Version 2004A) was employed. This software was used to generate the mean chromatogram as a representative standard fingerprint chromatogram for a group of chromatograms, calculate the correlation coefficients among these, and carry out a similarity analysis to compare the samples with the mean chromatogram based on the peak areas. The relative retention time (RRT) and relative peak area (RPA) of each common peak in the mean chromatogram were calculated, using calycosin (C) as reference within each preparation, respectively, to semi-quantitatively express the different chemical properties in the chromatographic profiles of the samples.

## Results and Discussion

### Method Optimization for the Analysis of Isoflavonoids

#### Optimization of Sample Preparation Methods for AR Pieces

The Chinese Pharmacopeia (National Commission of Chinese Pharmacopoeia, 2015) uses reflux heating for 4 h in methanol for the sample preparation method for the determination of CG and AG IV contents in AR roots. However, such a method is not convenient for analysis of a large number of samples. To shorten the time of sample preparation, we developed an ultrasonic extraction method. Different parameters were investigated, including the amount of MeOH, ultrasonication times, and extraction time, to reach a complete extraction of C. The reason why C instead of the CG was selected as the Q-marker of AR was demonstrated in *The Stability of Isoflavonoids in AR During Manufacturing Process* section. The amount of sample was kept the same as used in the Chinese Pharmacopeia (version 2015) (National Commission of Chinese Pharmacopoeia, 2015). The results indicated that the most efficient dissolution was sonication with methanol for three times (Supplementary Table S1), as described in *Sample Preparation for Isoflavonoid Analysis* section.

#### Optimization of the Sample Preparation Methods for Freeze-Dried Powder and Formula Granules of AR

Different sample preparation methods for freeze-dried powder and formula granules of AR were investigated. The amount of sample was calculated in accordance with the extraction rate (∼33.0%) and the proportion of its excipients in granules to ensure that the amount in the sample was consistent with that in the AR pieces. The extraction ability of different solvents (water, 5% methanol, and 50% methanol) and extraction times (10, 30, and 60 min) to extract C were tested. The results indicated that the most efficient solvent was the one using 5% methanol as solvent with sonication for 10 min (Supplementary Table S1), as described in *Preparation of Solutions* section.

#### Optimization of HPLC Separation

An HPLC-DAD fingerprint analysis of the isoflavonoids in AR was established based on the literature (Qi et al., 2008). The chromatographic conditions were further optimized based on the following principles: 1) to be suitable for the analysis of both the alcohol and the aqueous extract of AR; and 2) to be able to detect the main isoflavonoids in the extracts of AR with baseline separation. After optimizing different chromatographic parameters, including the elution gradient, column temperature, and injection volume, were set as described in *HPLC and MS Analysis Conditions* section.

### The Stability of Isoflavonoids in AR During Manufacturing Process

#### Fingerprint Analysis of Isoflavonoids

**FIGURE 2:**
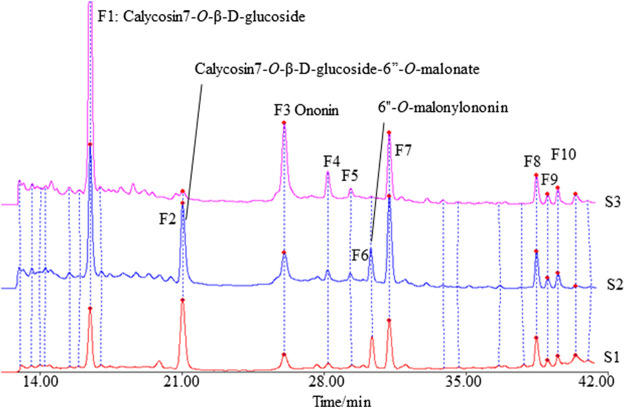
The mean chromatograms of isoflavonoids used as representative fingerprint chromatograms in analyses of the extracts of Astragali radix pieces (S1), freeze-dried powders (S2), and formula granules (S3) by HPLC-DAD at 260 nm [F1: calycosin 7-*O*-β-D-glucoside; F2: calycosin 7-*O*-β-D-glucoside-6″-*O*-malonate; F3: formononetin-7-*O*-glucoside; F4: (6α*R*, 11α*R*)-3-hydroxy-9,10-dimethoxypterocarpan-3-*O*-β-D-glucoside; F5: (3*R*)-7,2′-dihydroxy-3′,4′-dimethoxyisoflavan-7-*O*-β-D-glucoside; F6: 6″-*O*-malonylononin; F7: calycosin; F8: formononetin; F9: (6α*R*, 11α*R*)-3-hydroxy-9,10-dimethoxypterocarpan; F10: (3*R*)-7,2′-dihydroxy-3′,4′-dimethoxyisoflavan].

The fingerprints of 12 batches of AR pieces, 12 batches of freeze-dried AR powders, and six batches of AR formula granules, from the same manufacturer, were analyzed using the established conditions of fingerprint analysis. The mean chromatograms of AR pieces, freeze-dried powders, and formula granules were generated as described in *Data Analysis* section (Figure 2), and 10 common peaks were observed for AR among raw materials, intermediates, and final preparations. The common peaks were identified with high-resolution MS by comparisons with our in-house database, and confirmed with analyses of standard compounds. These common constituents included isoflavones [calycosin 7-*O*-β-D-glucoside (CG, F1), calycosin (C, F7), calycosin 7-*O*-β-D-(6″-malonyl)-glucoside (CGM, F2), formononetin-7-*O*-glucoside (FG, F3), formononetin (F, F8), and 6″-*O*-malonylononin (FGM, F6)], pterocarpans [(6α*R*, 11α*R*)-3-hydroxy-9,10-dimethoxypterocarpan-3-*O*-β-D-glucoside (F4) and (6α*R*, 11α*R*)-3-hydroxy-9,10-dimethoxypterocarpan (F9)], and isoflavans [(3*R*)-7,2′-dihydroxy-3′,4′-dimethoxyisoflavan-7-*O*-β-D-glucoside (F5) and (3*R*)-7,2′-dihydroxy-3′,4′-dimethoxyisoflavan (F10)] (Qi et al., 2008; Qi et al., 2009) (Table 2). The RRT and RPA of these 10 common peaks were calculated using C (F7) as the reference peak in the mean chromatogram of each respective preparation (Table 3).

**TABLE 2:** Assignment of identities to isoflavonoids and saponins detected in the HPLC chemical profiles of Astragali radix by high-resolution LC-MS.

No.^a^	Identification	Formula [M]	Experimental	Theoretical
F1	Calycosin 7-*O*-β-D-glucoside (CG)^b^	C_22_H_22_O_10_	[M + H, 447.1285]	447.1291
F2	Calycosin 7-*O*-β-D-(6″-malonyl)-glucoside (CGM)	C_25_H_24_O_13_	[M + H, 533.1246]	533.1295
F3	Ononin (FG)^b^	C_22_H_22_O_9_	[M + H, 431.1328]	431.1342
F4	(6α*R*,11α*R*)-3-hydroxyl-9,10-dimethoxypterocarpan-3-*O*-β-D-glucoside	C_23_H_26_O_10_	[M + H, 463.1585]	463.1604
F5	(3*R*)-2′7-dihydroxy-3′,4′-dimethoxyisoflavan-7-*O*-β-D-glucoside	C_23_H_28_O_10_	[M + H, 465.1748]	465.176
F6	6″-*O*-malonylononin (FGM)	C_25_H_24_O_12_	[M + H, 517.1364]	517.1346
F7	Calycosin (C)^b^	C_16_H_12_O_5_	[M + H, 285.07151]	285.0762
F8	Formononetin (F)^b^	C_16_H_12_O_4_	[M + H, 269.0886]	269.2812
F9	(6α*R*, 11α*R*)-3-hydroxy-9,10-dimethoxypterocarpan^b^	C_17_H_16_O_5_	[M + H, 301.1071]	301.1071
F10	(3*R*)-7,2′-dihydroxy-3′,4′-dimethoxyisoflavan^b^	C_17_H_18_O_5_	[M + H, 303.1239]	303.1232
S1	Astragaloside IV (Ag IV)^b^	C_41_H_68_O_14_	[M-H + FA-H, 829.4547]	829.4585
S3	Astragaloside Ⅱ (Ag Ⅱ)^b^	C_43_H_70_O_15_	[M-H + FA, 871.4705]	871.1691
S4	Isoastragaloside Ⅱ (iAg Ⅱ)^b^	C_43_H_70_O_15_	[M-H + FA, 871.4705]	871.1691
S5	Astragaloside I (Ag I)^b^	C_45_H_72_O_16_	[M-H + FA, 913.4780]	913.4796
S6	Isoastragaloside I (iAg I)^b^	C_45_H_72_O_16_	[M-H + FA, 913.4780]	913.4796
S7	Acetylastragaloside Ⅰ (AgA I)	C_47_H_74_O_17_	[M-H + FA, 955.4964] [M-H, 911.5013]	955.4902

a Refer to the same numbers in Figures 2, 3.

b Confirmed with standard compounds in HPLC-PDA.

**TABLE 3:** Relative retention time and relative peak area of common peaks in the mean chromatograms of pieces, freeze-dried powders, and formula granules of Astragali Radix with peak F7 in each chromatogram as reference.

No.^a^	Relative retention time	Relative peak area		
		Pieces	Freeze-dried powders	Formula granules
F1	0.53	1.14	1.61	4.74
F2	0.68	1.74	1.09	0.22
F3	0.84	0.44	0.54	1.74
F4	0.90	0.12	0.16	0.52
F5	0.94	0.06	0.13	0.28
F6	0.97	0.59	0.36	0.11
F7(s)	1.00	1.00	1.00	1.00
F8	1.23	0.59	0.36	0.38
F9	1.25	0.15	0.10	0.12
F10	1.26	0.25	0.15	0.21

a Refer to the same numbers in Figure 2.

#### Difference Between the Fingerprint of Isoflavonoids in Pieces, Freeze-Dried Powder, and Formula Granules of AR

The correlation coefficients between the mean chromatograms of the pieces, freeze-dried powder, and formula granules of AR showed that there were differences in the chemical compositions of the raw materials, intermediates, and final preparations of AR (Supplementary Table S2). Compared with AR pieces, the RPA of CG in freeze-dried powder and formula granules was significantly higher, while that of CGM was much lower. In formula granules, the content of CG was four times higher than that in other forms of AR, while the peak of CGM (F2) was almost absent. The same phenomenon was observed for FG and FGM. The chromatographic profiles of the aglycones (F8–F10) in the different preparations were similar.

#### The Thermal Stability of Isoflavonoids in the Water Decoction of AR

The remarkable increase in the RPA of CG and FG and the significant decrease in the RPA of CGM and FGM from raw materials to granules may be related to the instability of CGM and FGM. For further study, a decoction of AR was prepared and its thermal stability was investigated at 100°C. The results showed that the RPA of CGM and FGM decreased significantly after heating for 30 min, and after 4 h heating these peaks were not detectable anymore. Meanwhile, the RPA of CG and FG increased significantly with heating (Supplementary Figure S2). The duration of heating seems to be a major reason for the degradation of CGM and FGM into CG and FG in the aqueous decoction of AR. The levels of CG and FG in the final preparations of AR are thus dependent on the exposure time to heat in the manufacturing process. A previous study on FGM and CGM showed that these esters were almost completely converted into their related glycosides when the decoction was refluxed for 16 h in an 80% ethanol extract or stored in an 80% methanol extract at room temperature for 4 weeks (Lin et al., 2000). Apparently the levels of FGM and CGM seems to be good indicators for the processing of the aqueous and alcoholic preparations of AR roots, low levels of these compounds means that the material has been exposed to heat for prolonged time or has been stored for long periods.

At present, CG is used as the index for isoflavonoids in the quality evaluation of AR in the Chinese Pharmacopoeia (version 2015) (National Commission of Chinese Pharmacopoeia, 2015). However, due to the thermal instability of CGM, the content of CG will increase during sample preparation and storage. Therefore, although CG itself is stable during sample preparation, it is not suitable as a Q-marker of AR and its preparations. Our data show that C remains stable during the manufacturing process. A similar observation was reported for F in Hedysari radix (Liu et al., 2006). Calycosin has a variety of pharmacological properties, such as vascular endothelial cell protection, anti-osteoporosis, antioxidant, anti-tumor, and immunomodulating properties (Zhang et al., 2015). In addition, it is a typical phytoestrogen, with estrogen-like effects. Our previous studies found that the estrogen receptor activity of C is greater than that of CG. Therefore, C can be considered as a marker compound for AR because of its chemical properties as well as its activity. The sample preparation and determination methods of C were optimized as descried in *Method Optimization for the Analysis of Isoflavonoids* section and the final established methods were described in *Preparation of Solutions* and *HPLC and MS Analysis Conditions* sections.

### The Stability of Saponins in AR During Manufacturing Process

#### The Chemical Profile of Saponins in AR and Its Preparations

We first analyzed the saponins’ profile in AR by HPLC-ELSD (Figure 3A). A total of more than seven peaks were found to be in common for the different materials analyzed. By high-resolution MS they were identified as Astragaloside IV (AG IV), astragaloside III (AG III), astragaloside II (AG II), isoastragaloside II (iAG II), astragaloside I (AG I), isoastragaloside I (iAG I), and acetylAG I. A method for the simultaneous determination of the content of different saponins (AG IV, AG II, iAG II, AG I, iAG I, and acetyl AG I) was established using UPLC time-of-flight MS (UPLC-TOF-MS) (Qi et al., 2008). Analyzing the content of astragalosides in different preparations, the following observations were made: 1) in AR pieces, the content of AG I was significantly higher than that of other astragalosides; 2) in formula granules of AR, the content of AG II was significantly higher than that of other astragalosides; and 3) in the oral liquids of AR, AG IV was the main components. The astragalosides (AG I, iAG I, AG II, iAG II, and acetyl AG I) are acetylated derivatives of astragaloside IV, which differ only in the number and positions of the acetyl groups in their β-D-xylose attached at position C-3 (Supplementary Figure S1). The differences in the astragalosides’ profiles in the different preparations seem to be related to the loss of the acetyl groups of the xylose group during the manufacturing process. Loss of all acetyl groups results in AG IV as final product.

**FIGURE 3:**
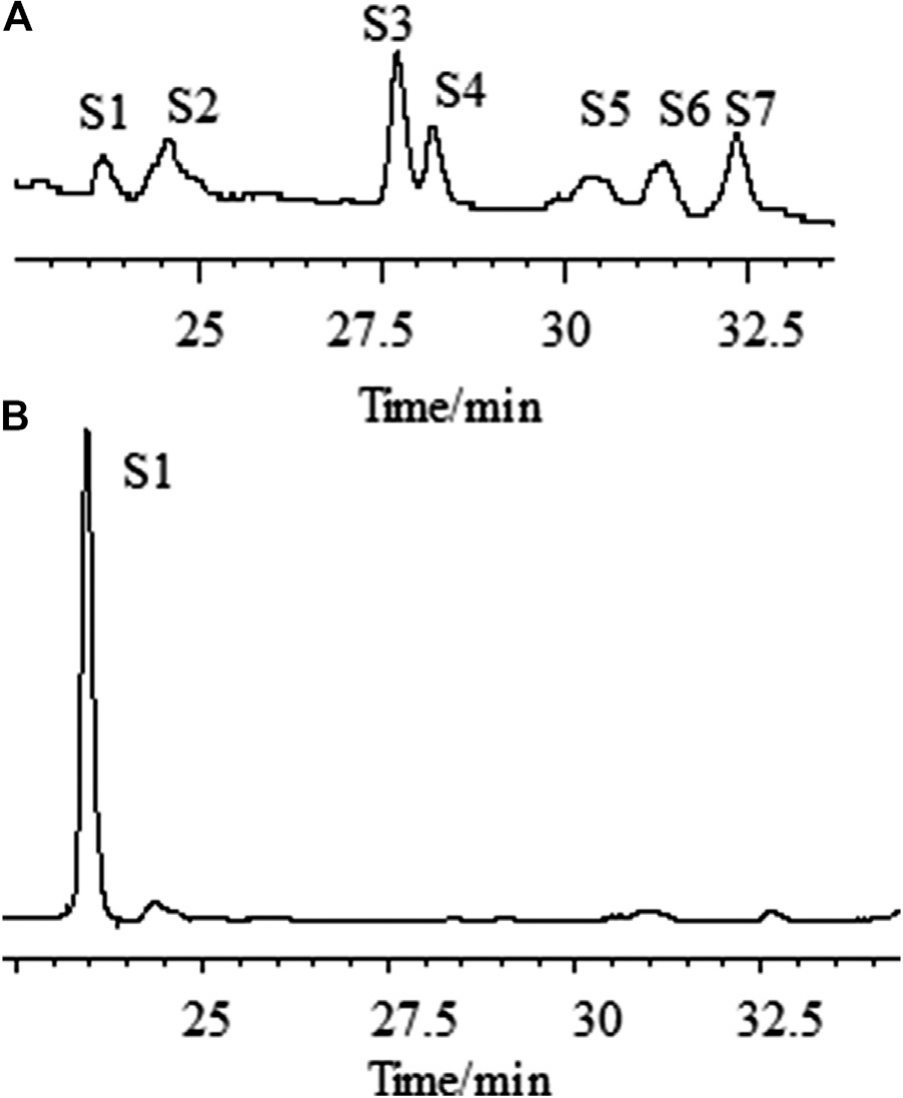
Chromatographic profiles of aqueous extracts of Astragali radix before **(A)** and after **(B)** the addition of ammonia, as determined by HPLC–ELSD. [S1: astragaloside IV (AG IV); S2: astragaloside III (AG III) and formononetin; S3: astragaloside II (AG II); S4: isoastragaloside II (iAG II); S5: astragaloside I (AG I); S6: isoastragaloside I (iAG I); S7: acetylastragaloside I.]

#### The Stability of Astragalosides in Aqueous Solution at High Temperature

A decoction of AR was prepared and its thermal stability was investigated at 100°C. The results showed that the RPA of AG I, iAG I and iAG II decreased significantly with heating, while the RPA of AG IV increased significantly (data not shown). This result indicates that AG I, iAG I and iAG II in the decoction of AR were unstable and they may be transformed into AG IV during heating. Then, the stability of an aqueous solution of a mixture of astragalosides (AG II, iAG II, AG I, and iAG I) was tested. The HPLC chromatograms of the samples heated for 2 h at 100°C showed that the levels of AG II, iAG II, AG I, and iAG I decreased while the content of AG IV increased (data not shown). Further investigations of the stability of two single standard compounds (AG II and AG IV) showed that in an aqueous solution, at room temperature, AG II was partly transformed into AG IV, and heating at 100°C further promoted the conversion of AG II into AG IV, while AG IV was stable in this condition (Supplementary Figure S3). Thus, heating is a major factor promoting the deacetylation reaction, leading to the transformation of astragalosides. The proposed chemical degradations of astragalosides were summarized in Supplementary Figure S4. This explains the different saponin profiles of AR preparations. Heat and long term storage lead to preparations with only AG IV, as found in oral liquid samples of AR (Qi et al., 2008). This implies that the saponins’ fingerprint for AR gives information about the manufacturing process and storage.

### Method Optimization for the Analysis of Astragaloside IV

The sample preparation for the AG IV determination in the Chinese Pharmacopoeia (National Commission of Chinese Pharmacopoeia, 2015) (version 2015) involves reflux extraction, liquid-liquid separation with *n*-butanol, and liquid-liquid separation with ammonia, which may take more than 2 days to complete per sample. Therefore, we optimized and established practical method for the determination of AG IV content in raw materials and pieces of AR (Zhao et al., 2018). This method replaces all of the time-consuming preparation steps with sonication extraction and ammonia hydrolysis, shortening the preparation time from around 2 days to less than 2 h. Sample preparation methods and analysis methods were optimized in this study for freeze-dried powder and formula granules of AR as described below.

#### Optimization of Extraction Methods for Freeze-Dried Powder and Formula Granules of AR

The sample preparation methods for freeze-dried powder and formula granules of AR were investigated. The extract ability of different solvents [5% methanol (v/v), 60% methanol (v/v), and methanol] and extraction times (10, 30, and 60 min) to extract AG IV were tested. The results indicated that extraction with 60% (v/v) methanol and sonication for 30 min showed the best performance in sample preparation (Supplementary Table S1), as described in *Sample Preparation for Isoflavonoid Analysis* section.

#### Optimization of the Amount of Ammonia

One critical step for the sample preparation of AR in Chinese Pharmacopoeia was reverse extraction with ammonia (National Commission of Chinese Pharmacopoeia, 2015). The purpose of this step was to transform other saponins into AG IV (Zhao et al., 2018). As an important parameter, the amount of ammonia solution needed for the transformation of astragalosides into AG IV was investigated. The fingerprint profiles of the astragalosides of AR extracts before and after adding 10 ml of ammonia solution ammonia were analyzed by HPLC-ELSD and compared (Figure 3). The results show that, under the specific conditions, the content of AG IV increased, while the peaks of all other astragalosides disappeared. The peak area of AG IV increased with the increased amount of ammonia solution, reaching its highest amount with 10 ml or more of ammonia solution. This implies that AG II, iAG II, AG I, iAG I, and acetyl AG I can be completely converted into AG IV which was stable under this alkaline conditions. Therefore, 10 ml of ammonia solution was used in this study.

#### Optimization of the Enrichment Methods of Astragaloside IV

The most practical method for the determination of AG IV content is the HPLC-ELSD. ELSD is a universal detector that gives very strong signals of primary compounds in the water extracts of herbal materials. Therefore it is necessary to remove the major primary metabolites from the aqueous AR extracts and the AR preparations, before the analysis of the saponins. Especially in case of formulated granules, the various excipients may interfere with the analysis. An SPE column separation method was applied to enrich AG IV in samples and remove the primary compounds. The following parameters were investigated in a step-by-step approach: the amount of ammonia added (1, 5, 10, 12, and 15 ml), the size of the SPE column (1,000 mg/6 ml and 500 mg/3 ml), the volume used in the water elution (3, 5, and 6 ml), and the volume used in the methanol elution (3, 5, and 6 ml). Finally, the content of AG IV was compared among procedures and the optimized methods were described in *HPLC-ELSD Conditions for Astragaloside Content Determination* section.

#### Optimization of ELSD Conditions for the Determination of Astragaloside IV

The effects of the elution gradient, carrier gas velocity (2.3, 2.4, 2.5, 2.7, and 2.8 L/min), and temperature of the drift tube (100, 105, and 110°C) of ELSD were investigated simultaneously. The largest peak area of AG IV was obtained at the flow rate of 2.4 L/min and the drift tube temperature of 110°C. The analytical method to determine AG IV content was described in *HPLC-ELSD Conditions for Astragaloside Content Determination* section.

### Method Validation and Sample Analysis

#### Method Validation

The calibration curve of C was successfully constructed as follows: Y = 90,909X − 19.877, where Y is the peak area and X is the concentration of C (mg/ml). The calibration curve of AG IV was also constructed, as: ln(Y) = 1.6723ln(X) − 1.8336, where ln(Y) is the natural logarithm of the peak area and ln(X) is the logarithm of the concentration of AG IV (mg/L). The LOD, LOQ, precision, stability, repeatability, and accuracy of the established methods for the determination of C and AG IV content are summarized in Supplementary Table S3. All results for the precision, stability, repeatability, and accuracy of these methods confirmed their validity and acceptability.

#### Sample Analysis

Samples of 12 batches of AR pieces, 12 batches of AR freeze-dried powders, and six batches of AR formula granules from the same manufacturer were analyzed by applying the specific extraction procedure for each type of material and the chromatographic methods for isoflavonoids and saponins. All analyses were repeated three times, and the data were recorded and expressed as the mean contents of C and AG IV (Table 1). The results demonstrated that this developed method was suitable for the determination of C and AG IV content in AR, from its raw materials to its final preparations.

## Conclusion

This study demonstrates that investigating the manufacturing process of traditional medicines from their raw materials to final preparations/products by chemical profile analysis is useful and effective for both the selection of suitable marker components and establishment of proper analytical methods for the quality control of traditional medicines. The quality control of the whole manufacturing process needs the process independent markers from the original raw plant material. For AR roots that is calycosin in case of the isoflavonoids and astragaloside IV in case of saponins. The established fingerprint methods for isoflavonoids and quantification methods for calycosin and AG IV were further validated by the analysis of a large number of samples, ranging from raw materials to final products. The developed methods were found to be simple, robust and reproducible, and thus suited as reliable quality control procedures for AR and its derived products. This work provides a demonstration study on the whole-process quality control of TCM and provides reference for improving the quality control methods of AR in Chinese Pharmacopoeia. The results suggest that it is necessary to reanalyze the quality control methods of other Chinese herbal medicines and their products derived from their water decoctions.

## Data Availability Statement

The raw data supporting the conclusions of this article will be made available by the authors, without undue reservation, to any qualified researcher.

## Author Contributions

YD, XQ, and SC designed the study. DW, MZ, LY, and PL did the experiments. YD, DW, and RV wrote the manuscript. All authors gave approval to the final version.

## Funding

This work was supported by the Natural Science Foundation (grant numbers: 81473340, 81803734) and Project of China Academy of Chinese Medical Sciences (grant numbers: ZXKT17009, GH201701).

## Conflict of Interest

The authors declare that the research was conducted in the absence of any commercial or financial relationships that could be construed as a potential conflict of interest.
