# Supplementary material for: Quality Markers for Astragali Radix and Its Products Based on Process Analysis
Source: Front Pharmacol. 2020 Dec 18;11:554777. doi: 10.3389/fphar.2020.554777 (PMC7775524; doi:10.3389/fphar.2020.554777)
Supplement: Supplementary file 1 [file datasheet1.docx]

***Supplementary Materials***

**Quality markers for Astragali radix and its products based on process analysis**

Yuntao Dai^a*#^, Dongbo Wang^a#^, Manjia Zhao^a^, Lihua Yan^a^, Chao Zhu^b^, Pengyue Li^a^, Xuemei Qin^c^, Rob Verpoorte ^d^, Shilin Chen^a*^

a Institute of Chinese Materia Medica, China Academy of Chinese Medical Sciences,100700, Beijing, China

b College of medicine and nursing, Dezhou University, 253023, Shandong, China

c China Modern Research Center for Traditional Chinese Medicine of Shanxi University, Shanxi University, Shanxi, China

d Natural Products Laboratory, Institute of Biology, Leiden University, Sylviusweg 72 2333BE Leiden, The Netherlands

* **Correspondence author**: Prof. Yuntao Dai ([ytdai@icmm.ac.cn](mailto:ytdai@icmm.ac.cn)), Prof. Shilin Chen (slchen@icmm.ac.cn)

# Co-first author

**1 Supplementary Figures**


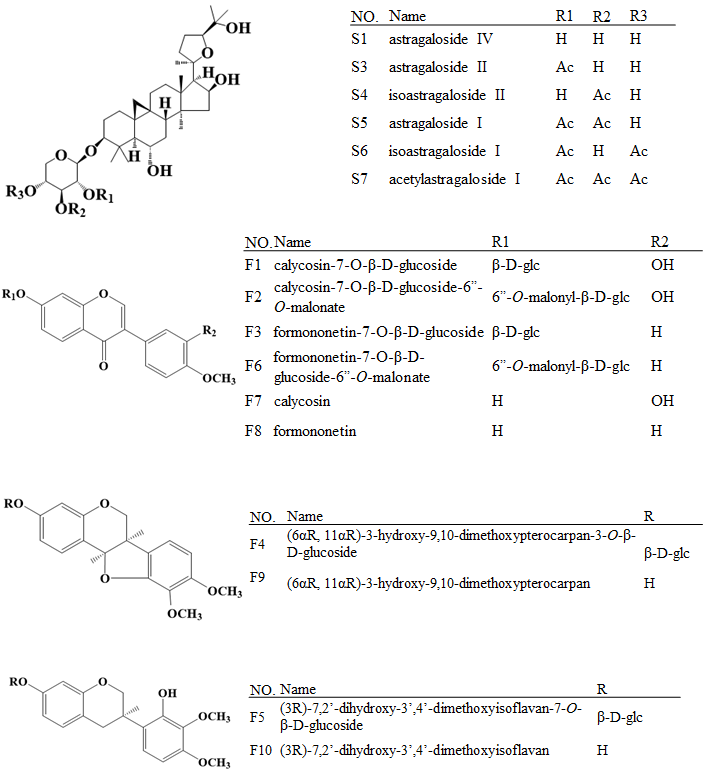


**Supplementary Figure 1.** Chemical structures of the major isoflavonoids and astragalosides in Astragali radix.


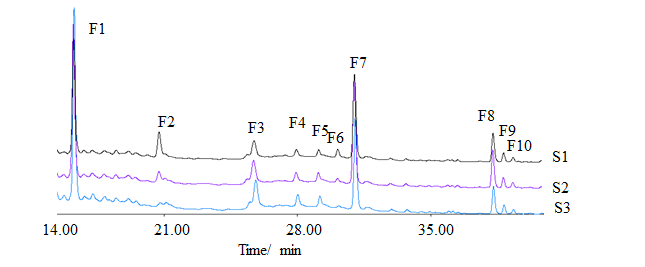


**Supplementary Figure 2.** HPLC chromatograms of the water extract of Astragali radix before and after heating. (S1, before heating; S2, heating at 100 ℃ for 30 min; S3, heating at 100 ℃ for 4 hours). [F1:Calycosin7-*O*-β-D-glucoside; F2: Calycosin7-*O*-β-D-glucoside-6”-*O*- malonate; F3: Fomononetin-7-*O*-glucoside; F4: (6α*R*, 11α*R*)- 3-hydroxy-9,10- dimethoxypterocarpan-3-*O*-β-D- glucoside; F5: (3*R*)-7,2’-dihydroxy-3’,4’- dimethoxyisoflavan-7-*O*-β-D-glucoside; F6: 6"-*O*-malonylononin; F7: Calycosin; F8: Formononetin; F9: (6α*R*, 11α *R*)-3-hydroxy-9,10-dimethoxypterocarpan; F10: (*3R*)- 7,2’-dihydroxy-3’,4’-dimethoxyisoflavan].


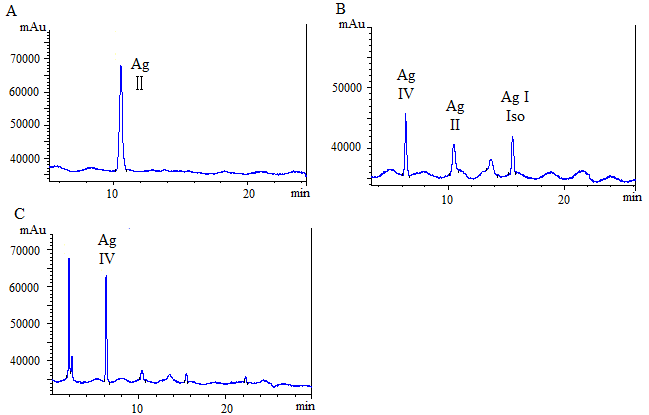
 **Supplementary Figure 3.** HPLC chromatograms of pure compound astragaloside II (AG II) in methanol (A), in water solution at room temperature (B) and after heating (C) at 100°C for 2 h detected by ELSD (evaporative light scattering detector). [AG IV: astragaloside IV; AG II: astragaloside II ; AG I Iso: astragaloside I isomer];


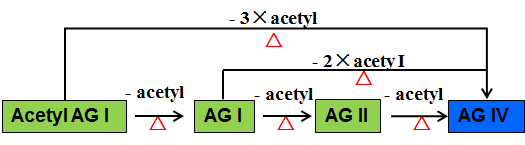


**Supplementary Figure 4.** The proposed chemical degradations of astragalosides during the manufacturing processing of AR due to high temperature (△).

**2 Supplementary Tables**

| Methanol concentration/% | Ultrasonic time/min | Peak area of calycosin  (the freeze-dried powders) | Peak area of calycosin  (the formula granules) |
| --- | --- | --- | --- |
| 5 | 10 | 483 | 2041 |
| 5 | 30 | 323 | 2182 |
| 5 | 60 | 288 | 2119 |
| 50 | 10 | 303 | --- |
| 100 | 10 | 286 | --- |
| 50 | 30 | --- | 1734 |
| 100 | 30 | --- | 1019 |

**Supplementary Table 1.** Optimization of the sample preparation of the freeze-dried powders and formula granules.

**Supplementary Table 2.** The correlation coefficients among the mean chromatograms of chopped plant material, freeze-dried powders, and granules of Astragali Radix shown in figure 2, calculated by the software “Similarity Evaluation System for Chromatographic Fingerprint of TCM”.

|  | Pieces | Freeze-dried powders | Formula  Granules |
| --- | --- | --- | --- |
| Pieces | 1 | 0.746 | 0.555 |
| Freeze-dried powders | 0.746 | 1 | 0.625 |
| Formula Granules | 0.555 | 0.625 | 1 |

**Supplementary Table 3** The calibration curves, Linearity range, precision, repeatability, stability, and accuracy for the determination of calycosin by HPLC-DAD and astragaloside IV by HPLC-ELAD in pieces, freeze-dried powders, and formula granules of Astragali Radix.

|  | calycosin | astragaloside IV |
| --- | --- | --- |
| Linear | Y= 90909X -19.877，r=0.9999 | ln(Y)= 1.6723ln(X)-1.8336，r=0.9995 |
| Linearity range（mg/ml） | 0.00055-0.055 | 0.27-0.54 |
| Precision RSD (%) (*n* = 6) | 0.34 | 3.72 |
| Repeatability RSD (%) (*n* = 6) | 0.29 | 1.16 |
| Stability RSD (%) (*n* = 6) | 2.18 | 2.41 |
| Recovery (%) (the pieces) (*n* = 6) | 104.45 (RSD 2.10%) | 100.88 (RSD 3.19%) |
| Recovery (%) (the freeze-dried powder) (*n* = 6) | 106.18 (RSD 1.50%) | 103.39 (RSD 3.87%) |
| Recovery (%) (the formula particle) (*n* = 6) | 96.55 (RSD 0.10%) | 98.47 (RSD 1.51%) |
